# A Rare Vascular Challenge: Brachial Artery Collateral Pseudoaneurysm Managed With Vein Grafting

**DOI:** 10.1155/cris/8844140

**Published:** 2025-09-26

**Authors:** Mario Malangone, Nunzio Montelione, Vincenzo Catanese, Francesco Alberto Codispoti, Andrea Cucci, Dalila Di Palma, Francesco Spinelli, Francesco Stilo

**Affiliations:** Department of Vascular Surgery, Policlinico Universitario Campus Bio-Medico, Rome, Italy

**Keywords:** collateral pseudoaneurysm, idiopathic pseudoaneurysm, infected false aneurysms, pseudoaneurysms of brachial artery, risks of endovascular procedures, upper extremity aneurysms

## Abstract

Upper extremity pseudoaneurysms are uncommon, and involvement of collateral branches of the brachial artery is particularly rare. A 78-year-old woman without antecedent trauma presented with a new pulsatile mass and progressive dysfunction of the proximal arm. Duplex ultrasonography provided sufficient diagnostic and planning information, demonstrating a pseudoaneurysm arising from a collateral branch of the brachial artery measuring 38 mm × 29 mm × 46 mm, with an estimated neck diameter of 3 mm and neck length of 5 mm, and a thin peripheral mural thrombus. Given lesion size and compressive features, open repair was performed under general anesthesia: sac excision and arterial reconstruction with a reversed basilic vein interposition graft using end-to-end anastomoses. The postoperative course was uneventful, with discharge on postoperative Day 1; at 7-day follow-up, duplex ultrasonography confirmed patency of the reconstructed segment without stenosis, residual sac, arteriovenous fistula, or signs of distal ischemia. This case supports open venous autologous reconstruction as an effective option for large and compressive arterial pseudoaneurysms of brachial collateral branches.

## 1. Introduction

Upper extremity arterial aneurysms are uncommon. In the Vietnam Vascular Registry, upper extremity pseudoaneurysms accounted for 27.4% of all recorded pseudoaneurysms, reflecting the trauma-dominant case mix of that cohort and providing an early large-scale estimate of anatomic distribution [1]. True aneurysms of the upper limb are exceedingly rare and are most often attributed to repetitive trauma, with the remainder considered idiopathic [2]. By contrast, pseudoaneurysms are pulsatile masses caused by a contained disruption of the arterial wall, with persistent communication to the parent vessel and a fibrous capsule; in the upper limb, they are usually iatrogenic after arterial puncture or percutaneous and endovascular procedures, may progress to rupture, infection, cutaneous ischemia, or compressive neuropathy, and are evaluated primarily with duplex ultrasonography [3, 4]; treatment aims to exclude the sac while preserving arterial continuity, using compression or percutaneous methods for small lesions and endovascular or open repair according to symptoms and anatomy [4].

This report describes an idiopathic pseudoaneurysm of a collateral branch of the brachial artery, occurring without antecedent contusion, trauma, or known infection, managed by sac excision and arterial reconstruction with an autologous vein interposition graft.

## 2. Case Report

A 78-year-old woman with polyneuropathy presented to our attention with a newly detected pulsatile swelling in the proximal third of the left arm, without antecedent local trauma. Her history included thoracic endovascular aortic repair (TEVAR) in 2017 for exclusion of a descending thoracic aortic aneurysm. Additional comorbidities were dyslipidemia and long-standing tobacco use (about 15 cigarets/day for 60 years). Prior surgeries included bilateral cataract extraction, right total knee arthroplasty, and right saphenectomy.

**Figure 1:**
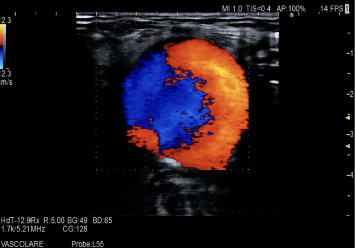
Elliptical pseudoaneurysm arising from a collateral branch, grayscale ultrasound.

**Figure 2:**
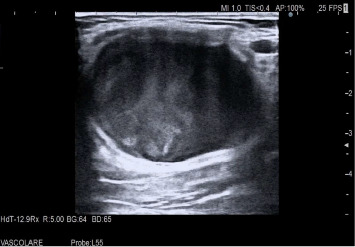
Color doppler ultrasound, “yin–yang” pattern.

At presentation, the medication regimen included telmisartan, amlodipine, clopidogrel, acetylsalicylic acid, primidone, levodopa/carbidopa, and folic acid. Vital signs were within normal limits; neurological findings were consistent with baseline polyneuropathy. The patient reported 30 days of progressive pain and functional limitation of the left upper limb. Duplex ultrasound examination demonstrated a pseudoaneurysm arising from a collateral branch of the brachial artery, measuring 38 mm × 29 mm × 46 mm (transverse × anteroposterior × craniocaudal), with a neck diameter of approximately 3 mm and neck length of approximately 5 mm; a thin peripheral mural thrombus occupied about 20% of the sac, and color doppler showed the typical “to-and-fro” flow at the neck (Figures 1,2). Evaluation of the deep and superficial venous systems revealed no deep venous thrombosis or superficial thrombophlebitis, excluding compressive sequelae and confirming suitability of an autologous venous conduit.

In view of these findings and the availability of an adequate ipsilateral basilic vein, the patient was scheduled for resection of the pseudoaneurysm and arterial reconstruction with autologous vein interposition. Approximately 10 days elapsed between assessment and surgery; clopidogrel was withheld, and acetylsalicylic acid was continued. Preoperative assessment assigned American Society of Anesthesiologists (ASA) physical status I and a low risk of venous thromboembolism.

**Figure 3:**
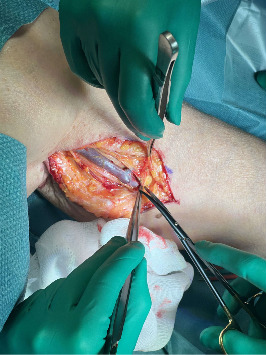
Harvested ipsilateral basilic vein.

**Figure 4:**
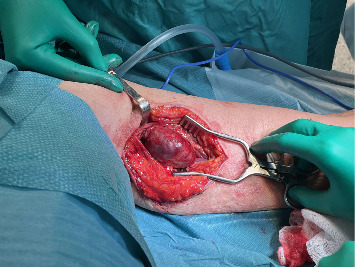
Intraoperative exposure of the lesion.

**Figure 5:**
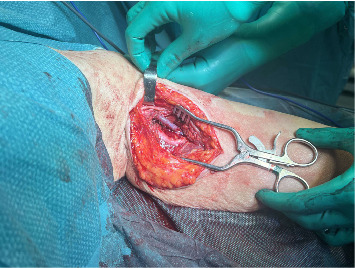
Successful vein graft anastomosis.

Under general anesthesia, prophylactic cefazolin (2 g) was administered pre-incision. A medial arm incision (about 8 cm) at the mid-brachium was made to expose the brachial artery collateral and the pseudoaneurysm. The basilic vein was harvested through the same incision and prepared as a reversed interposition graft. The median and ulnar nerves were identified and protected. Proximal and distal vascular control of the collateral branch was obtained, followed by aneurysmectomy. Arterial continuity was restored with a reversed basilic-vein interposition graft using end-to-end anastomoses (polypropylene 6–0 proximally and 7–0 distally). Intraoperative duplex confirmed laminar flow through the graft with palpable radial and ulnar pulses. A closed-suction drain was placed, and the wound was closed in layers (Figures 3–5).

The postoperative course was uneventful, with no residual motor or sensory deficit of the left upper limb; the patient was discharged on postoperative Day 1 in good clinical condition. At the 30-day follow-up, the patient was in good clinical condition, the left arm well perfused with a completed healed surgical incision. The duplex ultrasound examination, used for surveillance in accordance with recommendations that identify duplex as the first-line modality for evaluation and follow-up of peripheral pseudoaneurysms [3], demonstrated patency of the brachial artery and its collateral branch, without stenosis, and triphasic flow to the distal arteries of the arm.

## 3. Discussion

Pseudoaneurysms of the brachial artery are uncommon, and involvement of collateral branches has been reported only sporadically [5]. Most lesions are iatrogenic after arterial access or endovascular procedures, and additional cases follow blunt or penetrating trauma [5]. Less frequently, pseudoaneurysms arise in association with systemic arteriopathies, notably the vascular type of Ehlers–Danlos syndrome [6]. Sometimes the cause can not be identified, which is why we begin to talk about idiopathic pseudoaneurysms [2].

In vascular access using catheters, periprocedural complications include dilatation/pseudoaneurysm, hematoma, and thrombosis; risk is influenced by vascular status, antithrombotic therapy, arterial anatomy, access site and technique, first-pass success, and the quality of postprocedural compression. Complication rates are mitigated by careful patient selection, standardized technique with prompt manual compression, and routine ultrasound-guided cannulation [7, 8].

Therefore, ultrasound guided arterial cannulation is superior to the palpation or landmark technique, particularly for deep or small caliber targets and in obese or anticoagulated patients, because it provides real time visualization of calcified plaques and precise needle entry, thereby improving first pass success [8]. Although evidence at the brachial site is limited, studies at the femoral site show lower overall complication rates, fewer arteriovenous fistulas, and less procedure related pain with ultrasound guidance [8]. Nevertheless, in some cases after arterial puncture, duplex ultrasound should be used as they imaging modality to assess suspected pseudoaneurysm, and it can also support postoperative surveillance when appropriate [3].

For diagnostic purposes, the color duplex ultrasound is incredibly effective, capable of showing the classic “yin–yang” pathway of flow. Duplex ultrasound shows high diagnostic accuracy for pseudoaneurysm, with sensitivity of 94% and specificity of 97%. In this case, initial assessment with duplex ultrasonography provided sufficient anatomic and hemodynamic detail to define sac size, neck characteristics, and inflow–outflow, allowing treatment planning without additional type of imaging. In accordance with recommendations that identify duplex as the first-line modality for diagnosis and follow-up of peripheral pseudoaneurysms [3], CT angiography was intentionally deferred, thereby improving cost–benefit and avoiding exposure to ionizing radiation and iodinated contrast without compromising clinical decision making.

Because the brachial, ulnar, and radial arteries are small in caliber, aneurysms in these vessels are uncommon and surgical exposure is straightforward; consequently, open repair remains the principal treatment. Nonoperative management of selected pseudoaneurysms has yielded favorable early and intermediate term outcomes: compared with femoral pseudoaneurysms, percutaneous thrombin injection in the upper-extremity arteries is technically more challenging because of smaller luminal diameter and a greater risk of distal embolization, which necessitates careful case selection [9, 10].

Small (<1.8–2.0 cm), asymptomatic pseudoaneurysms without concomitant antithrombotic therapy and with an identifiable neck may be observed or treated with manual/ultrasound-guided compression; failure of conservative measures may be followed by ultrasound-guided thrombin injection in appropriately selected patients (no prior bovine thrombin exposure or hypersensitivity) [5]. Endovascular stent-grafting or coil embolization is appropriate for small, distal branch pseudoaneurysms when collateral circulation is adequate, and landing zones are favorable. Open surgical repair is indicated for large (>2 cm) or infected lesions, rapidly expanding pseudoaneurysms, compressive syndromes (neuropathy and limb impairment/claudication), critical limb/skin ischemia, venous thrombosis, or when percutaneous options fail or anatomy is unsuitable [5].

In the present case, given lesion size with compression of the brachial artery and median nerve causing functional deficit, open repair was undertaken; stent placement was deemed inappropriate owing to the absence of a suitable landing segment.

## 4. Conclusion

Pseudoaneurysms of the brachial artery and its collateral branches are uncommon peripheral arterial dilatations, and the evidence base remains limited because of their low incidence. Etiology is heterogeneous and includes traumatic, infectious, idiopathic, and iatrogenic causes; patients scheduled for endovascular procedures should be counseled about this potential risk. Early diagnosis is crucial to plan therapy before rupture, and the assessment can be achieved with color doppler ultrasonography. Management is individualized according to size, location, thrombotic burden, and clinical presentation. In practice, open surgical repair is the preferred option for large or complicated lesions and is associated with durable outcomes in the medium and long term.

## Data Availability

The data that support the findings of this study are available on request from the corresponding author. The data are not publicly available due to privacy or ethical restrictions.

## References

[1] Ho P. K., Weiland A. J., McClinton M. A., Wilgis E. F. S. (1987). Aneurysms of the Upper Extremity. *The Journal of Hand Surgery*.

[2] Clark E. T., Mass D. P., Bassiouny H. S., Zarins C. K., Gewertz B. L. (1991). True Aneurysmal Disease in the Hand and Upper Extremity. *Annals of Vascular Surgery*.

[3] Coughlin B. F., Paushter D. M. (1988). Peripheral Pseudoaneurysms: Evaluation with Duplex US. *Radiology*.

[4] Saad N. E. A., Saad W. E. A., Davies M. G., Waldman D. L., Fultz P. J., Rubens D. J. (2005). Pseudoaneurysms and the Role of Minimally Invasive Techniques in Their Management. *RadioGraphics*.

[5] Borghese O., Pisani A., di Centa I. (2019). Treatment of Iatrogenic Pseudoaneurysm of the Brachial Artery: Case Report and Literature Review. *Vascular Disease Management*.

[6] Barabas A. P. (1972). Vascular Complications in the Ehlers-Danlos Syndrome, With Special Reference to the “Arterial Type” or Sack’s Syndrome. *The Journal of Cardiovascular Surgery*.

[7] Katzenschlager R., Ugurluoglu A., Ahmadi A. (1995). Incidence of Pseudoaneurysm After Diagnostic and Therapeutic Angiography. *Radiology*.

[8] Sobolev M., Slovut D. P., Lee Chang A., Shiloah A. L., Eisen L. A. (2015). Ultrasound-Guided Catheterization of the Femoral Artery: A Systematic Review and Meta-Analysis of Randomized Controlled Trials. *The Journal of Invasive Cardiology*.

[9] Sheiman R. G., Brophy D. P., Perry L. J., Akbari C. (1999). Thrombin Injection for the Repair of Brachial Artery Pseudoaneurysms. *American Journal of Roentgenology*.

[10] Buda S. J., Johanning J. M. (2005). Brachial, Radial, and Ulnar Arteries in the Endovascular Era: Choice of Intervention. *Seminars in Vascular Surgery*.

